# Flufenamic Acid, a Promising Agent for the Sensitization of Colistin-Resistant Gram-Negative Bacteria to Colistin

**DOI:** 10.1128/spectrum.04052-22

**Published:** 2023-03-27

**Authors:** Yi Zhang, Yijia Han, Lingbo Wang, Jingchun Kong, Wei Pan, Xiaodong Zhang, Lijiang Chen, Zhuocheng Yao, Tieli Zhou, Jianming Cao

**Affiliations:** a Department of Medical Lab Science, School of Laboratory Medicine and Life Science, Wenzhou Medical University, Wenzhou, Zhejiang Province, China; b Department of Clinical Laboratory, The First Affiliated Hospital of Wenzhou Medical University, Key Laboratory of Clinical Laboratory Diagnosis and Translational Research of Zhejiang Province, Wenzhou, Zhejiang Province, China; Universitat Greifswald

**Keywords:** combination, Gram-negative bacteria, colistin resistance, synergistic effect, multidrug resistance, antibiofilm, flufenamic acid

## Abstract

The continuous development of multidrug-resistant (MDR) Gram-negative bacteria poses a serious risk to public health on a worldwide scale. Colistin is used as the last-line antibiotic for the treatment of MDR pathogens, and colistin-resistant (COL-R) bacterial emergence thus has the potential to have a severe adverse impact on patient outcomes. In this study, synergistic activity was observed when colistin and flufenamic acid (FFA) were combined and used for the *in vitro* treatment of clinical COL-R Pseudomonas aeruginosa, Escherichia coli, Klebsiella pneumoniae, and Acinetobacter baumannii strains, as shown by checkerboard and time-kill assays. Crystal violet staining and scanning electron microscopy revealed the synergistic action of colistin-FFA against biofilms. When used to treat murine RAW264.7 macrophages, this combination did not induce any adverse toxicity. Strikingly, the survival rates of bacterially infected Galleria mellonella larvae were improved by such combination treatment, which was also sufficient to reduce the measured bacterial loads in a murine thigh infection model. Mechanistic propidium iodide (PI) staining analysis further demonstrated the ability of these agents to alter bacterial permeability in a manner that enhanced the efficacy of colistin treatment. Together, these data thus demonstrate that colistin and FFA can be synergistically combined to combat the spread of COL-R Gram-negative bacteria, providing a promising therapeutic tool with the potential to protect against COL-R bacterial infections and improve patient outcomes.

**IMPORTANCE** Colistin is a last-line antibiotic used for the treatment of MDR Gram-negative bacterial infections. However, increasing resistance to it has been observed during clinical treatment. In this work, we assessed the efficacy of the combination of colistin and FFA for the treatment of COL-R bacterial isolates, demonstrating that the combined treatment has effective antibacterial and antibiofilm activities. Due to its low cytotoxicity and good therapeutic effects *in vitro*, the colistin-FFA combination may be a potential candidate for research into a resistance-modifying agent to combat infections caused by COL-R Gram-negative bacteria.

## INTRODUCTION

The rapid emergence of multidrug-resistant (MDR) bacteria due to the widespread and improper use of antibiotics poses a serious threat to global public health (1). The use of colistin (COL) (polymyxin E) as an antibiotic of last resort for the clinical treatment of patients suffering from carbapenem-resistant Gram-negative bacterial infections has increased in recent years (2). However, excessive colistin use has been driving the increasingly rapid development of colistin-resistant (COL-R) bacteria for which few effective antimicrobial tools are available (3). High drug development costs have hampered the design of novel antibiotics (4). As a result, novel approaches capable of sensitizing bacteria to extant antibiotics are desperately needed to combat the rapid emergence and spread of antimicrobial resistance mechanisms (5). Screenings have focused on drug repurposing, and synergistic combinations of drugs represent a promising means of establishing new therapeutic regimens for treating drug-resistant bacteria and biofilms (6). These approaches can also help to reduce clinical trial dropout rates as a consequence of drug-related toxicity or poor pharmacokinetic properties while enhancing the antimicrobial efficacy of conventional antibiotics (7).

Biofilms are communities of bacteria embedded in a three-dimensional (3D) matrix of extracellular polymeric substances that enable these microbes to persist in harsh environments (8). These biofilms can shield the bacteria therein from antibiotic penetration and alter microenvironmental characteristics or bacterial genotypes, thereby driving the development of more robust antimicrobial responses and the onset of recurrent chronic infections (9, 10). Classical antibiotics characterized to date are not able to effectively facilitate biofilm eradication, underscoring the need to develop new treatment strategies capable of preventing the formation of these biofilms and/or eradicating mature established biofilms.

Flufenamic acid (FFA), a nonsteroidal anti-inflammatory drug (NSAID), is frequently used for the treatment of rheumatic conditions (11). Furthermore, FFA has also been reported to suppress the growth of Candida albicans, Salmonella enterica serovar Typhimurium, and methicillin-resistant Staphylococcus aureus (MRSA) and to prevent the establishment of MRSA biofilms (12–14). To date, there has been no investigation into the possibility of synergistic antimicrobial and antibiofilm activities between colistin and FFA when used to treat infections by COL-R Gram-negative bacterial clinical isolates such as Pseudomonas aeruginosa, Escherichia coli, Klebsiella pneumoniae, and Acinetobacter baumannii.

This study sought to examine whether colistin and FFA exhibit synergistic antimicrobial and antibiofilm activities when used to treat COL-R Gram-negative bacterial infections using multiple *in vitro* and *in vivo* analyses, thereby providing a foundation for future efforts to treat infections caused by COL-R pathogens.

## RESULTS

### Analyses of antimicrobial resistance profiles.

The MIC values of common clinical antibiotics used to treat specific bacterial isolates are summarized in Table 1. The clinical data for the patients and the characteristics of the analyzed strains are listed in Table S1 in the supplemental material. The majority of these bacterial isolates (28/31; 90.3%) exhibited MDR phenotypes against β-lactams, quinolones, and aminoglycosides. The isolates showed various levels of resistance to colistin and FFA at MIC values of >512 μg/mL, consistent with the lack of any intrinsic FFA-mediated antimicrobial activity when used to treat these tested COL-R Gram-negative bacteria.

**TABLE 1:** MIC values of common clinical antibiotics and FFA when used to treat COL-R Gram-negative bacteria^*c*^

	MIC (μg/mL)									
	Antibiotic at the indicated MIC breakpoints (μg/mL) (S–R)^*b*^									
	ATM	CAZ	FEP	IPM	CIP	LVX	GEN	TOB	COL	
Strain^*a*^	8–32	8–32	8–32	2–8	0.5–2	1–4	4–16	4–16	2–4	FFA
QC										
ATCC 25922	0.06	0.12	0.016	0.06	0.008	0.008	0.5	1	1	
ATCC 27853	4	1	2	1	0.25	0.5	1	1	1	
P. aeruginosa										
TL1671	8	4	8	2	0.25	1	2	1	**32**	>512
**TL1736**	4	4	2	**16**	1	1	**32**	8	**8**	>512
**TL1744**	**32**	**32**	16	**16**	**32**	**8**	**≥128**	**32**	**4**	>512
TL2314	16	32	16	4	0.5	2	8	2	**8**	>512
**TL2917**	**32**	16	16	**16**	0.25	2	8	8	**8**	>512
**TL2967**	**128**	16	**32**	**16**	**8**	**16**	8	8	**4**	>512
**TL3008**	4	2	4	**16**	0.5	1	**16**	4	**32**	>512
**TL3086**	**128**	16	16	**≥128**	**16**	**8**	**≥128**	**128**	**32**	>512
	4–16	4–16	2–16	1–4	0.25–1	0.5–2	4–16	4–16	2–4	
E. coli										
**DC90**	**≥256**	**32**	**64**	**≥256**	**64**	**32**	**≥256**	**128**	**8**	>512
**DC3539**	**64**	**128**	**≥128**	0.5	**64**	**16**	**128**	**128**	**8**	>512
**DC3599**	**64**	**16**	8	1	**4**	**8**	**16**	8	**8**	>512
**DC3806**	**64**	**64**	**16**	1	**4**	**8**	**16**	**16**	**8**	>512
**DC3846**	**128**	**64**	**≥256**	0.5	**≥256**	**128**	**≥256**	**64**	**8**	>512
**DC4887**	1	4	**32**	1	**4**	**16**	**16**	8	**8**	>512
**DC5286**	**≥256**	**128**	**≥256**	0.25	**128**	**64**	4	4	**8**	>512
**DC7333**	**≥256**	**≥256**	**≥256**	**16**	**≥256**	**128**	**128**	**≥256**	**4**	>512

K. pneumoniae										
**FK169**	**≥128**	**32**	**32**	0.25	**32**	**16**	2	2	**8**	>512
**FK1342**	**≥128**	**≥128**	**≥128**	0.25	**1**	0.5	1	4	**≥128**	>512
**FK1913**	**≥128**	**≥128**	**≥128**	**32**	**≥128**	**128**	**≥128**	**≥128**	**64**	>512
FK1986	0.0125	0.25	0.0125	0.25	0.0125	0.025	2	1	8	>512
**FK3810**	0.0125	**128**	**≥256**	**32**	**≥256**	**128**	**≥256**	**≥256**	**64**	>512
**FK3994**	**≥256**	**128**	**≥256**	**32**	**≥256**	**64**	**≥256**	**≥256**	**64**	>512
**FK6556**	**64**	**64**	**64**	**16**	**4**	**8**	**16**	**16**	**32**	>512
**FK6663**	**≥256**	**≥256**	**≥256**	**32**	**≥256**	**≥256**	**≥256**	**≥256**	**16**	>512
	—	8–32	8–32	2–8	1–4	2–8	4–16	4–16	2–4	
A. baumannii										
**BM1539**	16	8	**64**	**16**	**4**	2	1	1	**16**	>512
**BM1595**	2	**32**	8	4	**64**	**8**	**128**	**128**	**4**	>512
**BM2349**	16	**64**	**64**	**16**	**4**	**8**	1	1	**4**	>512
**BM2370**	8	**32**	**128**	**8**	**128**	**8**	4	1	**8**	>512
**BM2412**	16	**64**	**64**	**16**	**4**	**8**	4	1	**16**	>512
**BM2431**	64	**64**	**64**	**16**	**4**	**8**	1	1	**32**	>512
**BM2622**	64	**64**	**64**	**8**	**4**	**8**	**16**	**16**	**4**	>512

a Strains in boldface type are multidrug-resistant (MDR) strains.

b Values in boldface type indicate resistance. S–R represents the susceptible (S) breakpoint to the resistant (R) breakpoint, according to CLSI supplement M100 (34) and EUCAST (49).

c QC, quality control; ATM, aztreonam; CAZ, ceftazidime; FEP, cefepime; IMP, imipenem; CIP, ciprofloxacin; LVX, levofloxacin; GEN, gentamicin; TOB, tobramycin, COL, colistin; FFA, flufenamic acid.

### Checkerboard assays.

Checkerboard assays revealed that the MIC values of colistin were decreased by 4- to 512-fold across the tested bacterial isolates when combined with FFA (Table 2). With the exception of P. aeruginosa TL1736, TL1744, and TL2967 (fractional inhibitory concentration index [FICI] values of 2), the FICI values for the combinations of colistin and FFA ranged from 0.0175 to 0.375 across all tested COL-R bacterial strains, consistent with robust synergistic activity such that FFA potentiates the activity of colistin. The majority of the isolates (22/31) regained colistin susceptibility in the presence of FFA.

**TABLE 2:** FICI values for combinations of flufenamic acid and colistin used to treat COL-R Gram-negative bacteria^*a*^

Isolate	MIC (μg/mL)				FICI	Type of interaction
	Monotherapy		Combination therapy			
	COL	FFA	COL	FFA		
P. aeruginosa						
TL1671	32	>512	1	32	0.094	Synergistic
TL1736	8	>512	8	>512	2	No interaction
TL1744	4	>512	4	>512	2	No interaction
TL2314	8	>512	0.5	32	0.1875	Synergistic
TL2917	8	>512	1	8	0.1406	Synergistic
TL2967	4	>512	4	>512	2	No interaction
TL3008	32	>512	1	8	0.0469	Synergistic
TL3086	32	>512	4	64	0.25	Synergistic
E. coli						
DC90	8	>512	1	64	0.25	Synergistic
DC3539	8	>512	1	64	0.25	Synergistic
DC3599	8	>512	1	64	0.25	Synergistic
DC3806	8	>512	1	64	0.25	Synergistic
DC3846	8	>512	1	64	0.25	Synergistic
DC4887	8	>512	1	64	0.25	Synergistic
DC5286	8	>512	1	32	0.1875	Synergistic
DC7333	4	>512	1	64	0.375	Synergistic
K. pneumoniae						
FK169	8	>512	2	16	0.2813	Synergistic
FK1342	≥128	>512	8	64	0.1875	Synergistic
FK1913	64	>512	0. 5	16	0.0391	Synergistic
FK1986	8	>512	0.125	8	0.0313	Synergistic
FK3810	64	>512	0.125	8	0.0175	Synergistic
FK3994	64	>512	2	32	0.0938	Synergistic
FK6556	32	>512	2	64	0.1875	Synergistic
FK6663	16	>512	2	64	0.25	Synergistic
A. baumannii						
BM1539	16	>512	0.125	8	0.0234	Synergistic
BM1595	4	>512	0.125	8	0.0469	Synergistic
BM2349	4	>512	0.125	8	0.0469	Synergistic
BM2370	8	>512	0.125	8	0.1875	Synergistic
BM2412	16	>512	0.125	8	0.0234	Synergistic
BM2431	32	>512	0.125	8	0.0273	Synergistic
BM2622	4	>512	0.125	8	0.0469	Synergistic

a FICI, fractional inhibitory concentration index.

### Time-kill assays.

**FIG 1:**
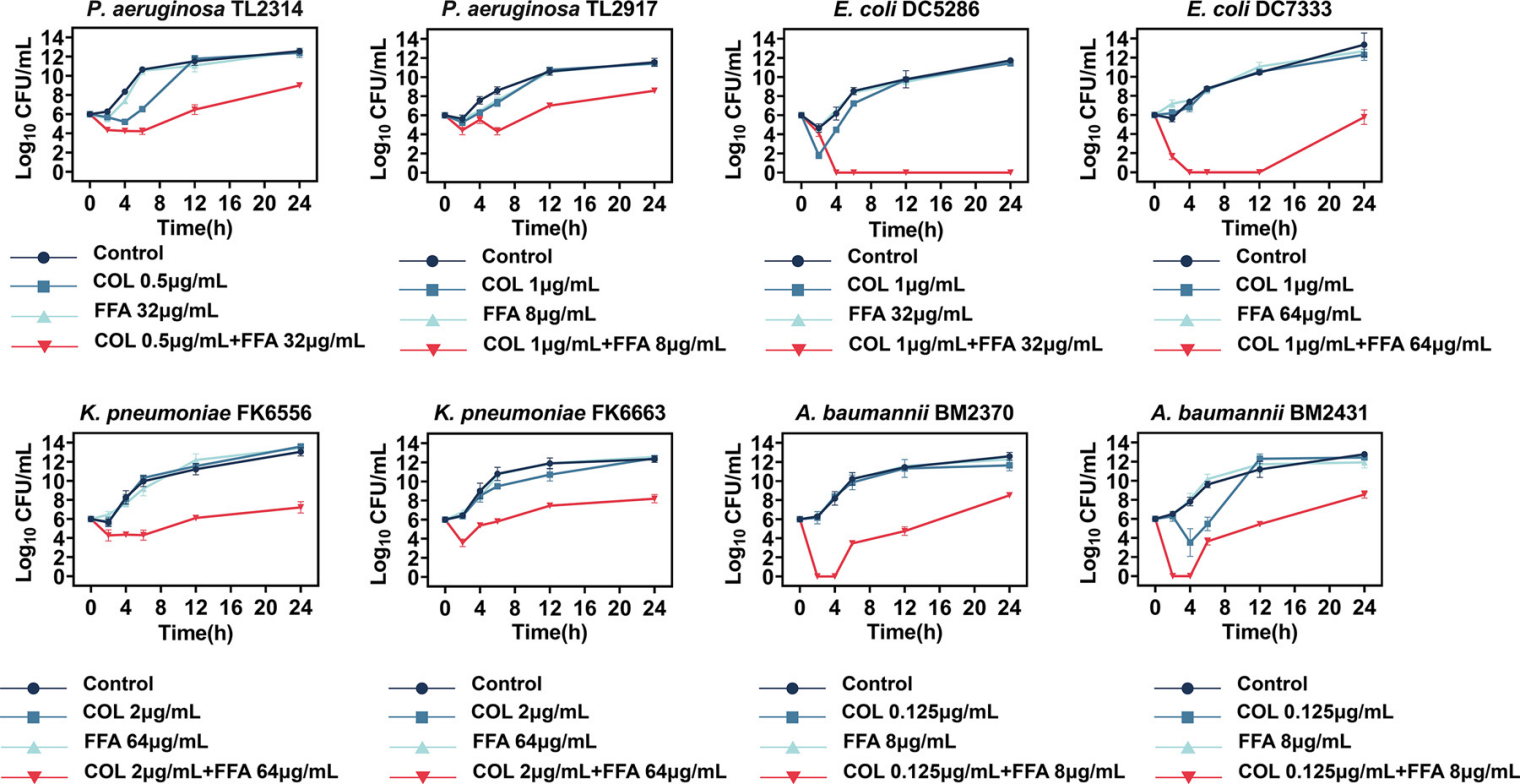
Time-kill curve results for colistin (COL) and flufenamic acid (FFA) alone or in combination against COL-R Gram-negative bacteria.

Time-kill assays were carried out for the further investigation of the synergy between colistin and FFA using eight randomly selected bacterial isolates (P. aeruginosa TL2314 and TL2917, E. coli DC5286 and DC7333, K. pneumoniae FK6556 and FK6663, and A. baumannii BM2370 and BM2431) (Fig. 1). Combinations of FFA and colistin were tested, with the selection of the optimal concentrations being based on the results of the checkerboard experiment (Table 2). The combination treatment effectively suppressed bacterial growth, reducing the number of viable bacterial cells by over 3 log_10_ CFU/mL over a 24-h period. These findings thus confirmed the bactericidal activity of colistin when combined with FFA.

### Impact on the formation and eradication of biofilms.

**FIG 2:**
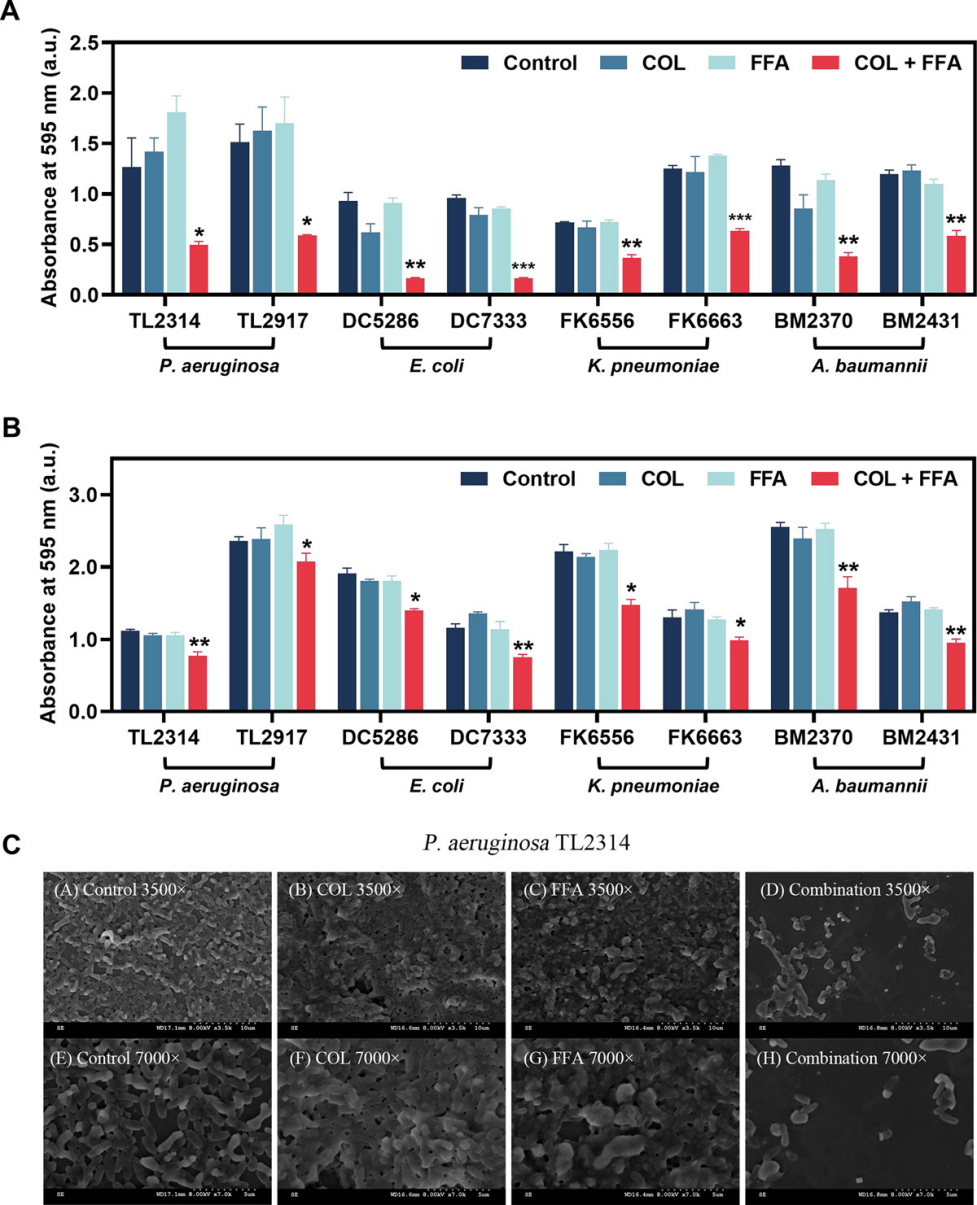
(A) Inhibition of biofilm formation by COL-R Gram-negative bacteria after treatment with colistin and/or FFA. (B) Eradication of COL-R Gram-negative bacterial biofilms by colistin and/or FFA. *, *P* < 0.05; **, *P* < 0.01; ***, *P* < 0.001 (by Student’s *t* tests). Data are means ± SD from triplicate analyses. (C) SEM images of colistin- and/or FFA-treated biofilms. a.u., arbitrary units.

The effects of colistin and/or FFA treatment on biofilm establishment or the ability to eradicate established biofilms composed of COL-R Gram-negative bacteria were next assessed by a crystal violet staining approach. The combination of FFA and colistin at subinhibitory concentrations (sub-MICs) determined by the checkerboard assay were able to significantly suppress biofilm formation by the tested bacterial strains (Fig. 2A), doing so more effectively than single-agent FFA or colistin treatment in all cases (*P < *0.05). The 595-nm absorbances of all eight strains were reduced by one-half, including that of P. aeruginosa, which has strong biofilm-forming abilities (15). The combination of 8 μg/mL colistin and 128 μg/mL FFA also readily eradicated established mature biofilms produced by all of the tested strains (Fig. 2B), compared with the control and monotherapy groups (*P < *0.05). Altogether, the combination of colistin and FFA can thus interfere with the ability of COL-R Gram-negative bacteria to form biofilms while also enabling the eradication of mature biofilms.

### Scanning electron microscopy analyses.

Scanning electron microscopy (SEM) analyses were next used to more thoroughly examine the effect of combination FFA-colistin treatment on biofilm formation (Fig. 2C). Untreated P. aeruginosa TL2314 cells exhibited a thick biofilm covering the entire visual field and interwoven with regularly shaped bacteria, and a similar densely organized biofilm was observed after monotherapy with colistin (0.5 μg/mL) and FFA (32 μg/mL). Colistin-FFA treatment in combination, in contrast, led to significant reductions in cell numbers together with reduced biofilm numbers and densities and only a small amount of bacterial aggregation. High-magnification (×7,000) imaging further confirmed the disruption of the original, complete, and tightly adherent biofilm, while visible bacterial cells showed morphological changes, including reductions in size and the release of cellular debris, together with the appearance of vesicles, bulges, and wrinkles.

### *In vitro* cytotoxicity analyses.

RAW264.7 cells were next used as a model mammalian cell line to detect any potential FFA-associated cytotoxicity. However, no increases in RAW264.7 cell death were observed following treatment with high-dose FFA (128 μg/mL), relative to the dimethyl sulfoxide (DMSO) or control treatment (Fig. S1). The combination regimen developed in this study thus offers an excellent *in vivo* safety profile.

### Analyses of *in vivo* therapeutic efficacy.

**FIG 3:**
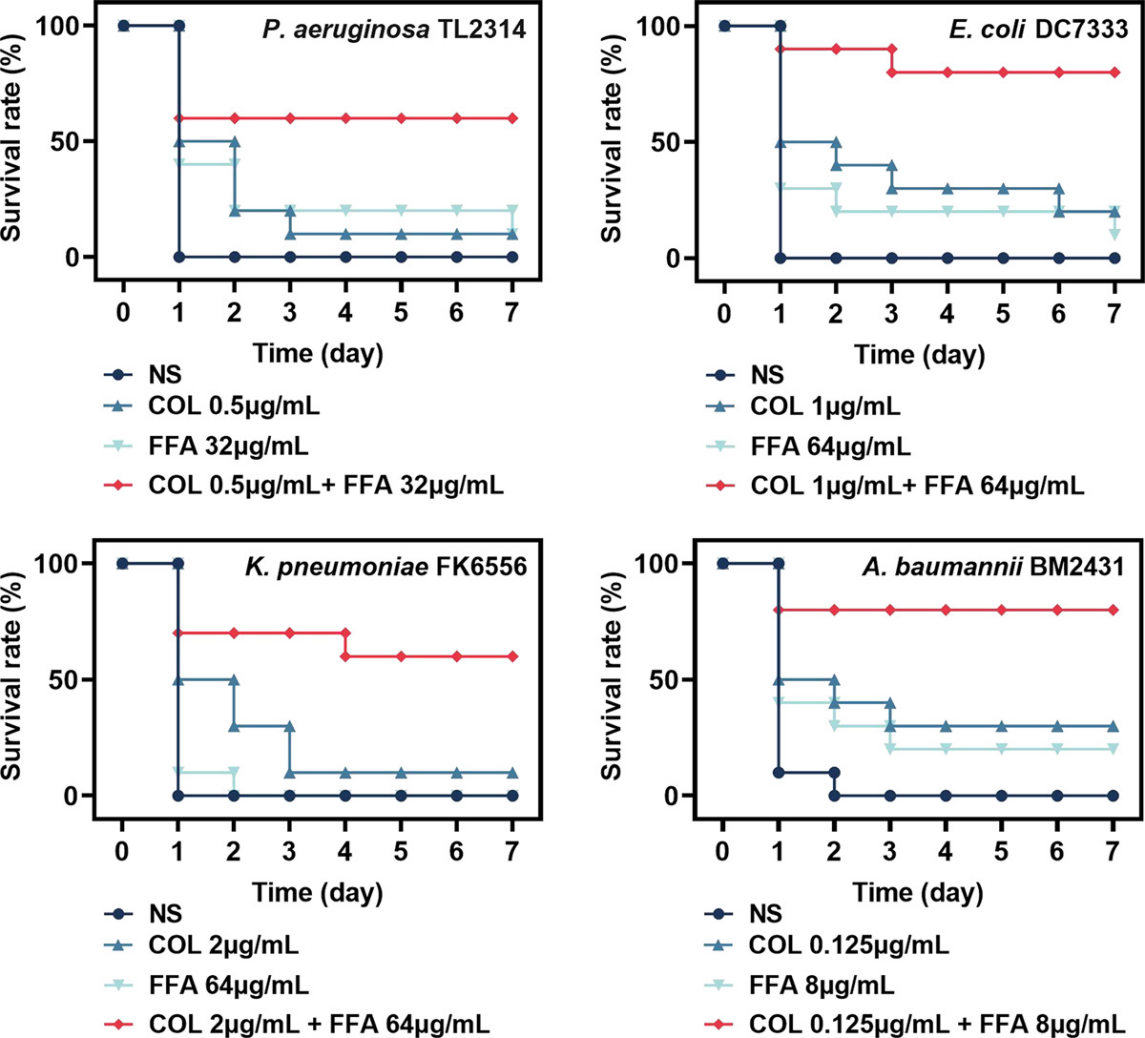
Galleria mellonella survival rates following a 7-day treatment period with a range of flufenamic acid (FFA) and/or colistin (COL) concentrations in larvae infected with COL-R P. aeruginosa TL2314, E. coli DC7333, K. pneumoniae FK6556, or A. baumannii BM2431 NS, normal saline.

The survival of Galleria mellonella larvae was next analyzed to gauge the *in vivo* synergistic benefits of FFA and colistin treatment in the context of COL-R bacterial infections. The TL2314, DC7333, FK6556, and BM2431 isolates were selected as targets for this experiment. While almost all untreated G. mellonella larvae died within 48 h following infection (Fig. 3), the combination treatment significantly improved the G. mellonella survival rates relative to the control group (*P <* 0.05) such that the rates of survival of P. aeruginosa- and K. pneumoniae-infected larvae increased by 60% and those of E. coli- and A. baumannii-infected larvae increased to 80% after 168 h.

**FIG 4:**
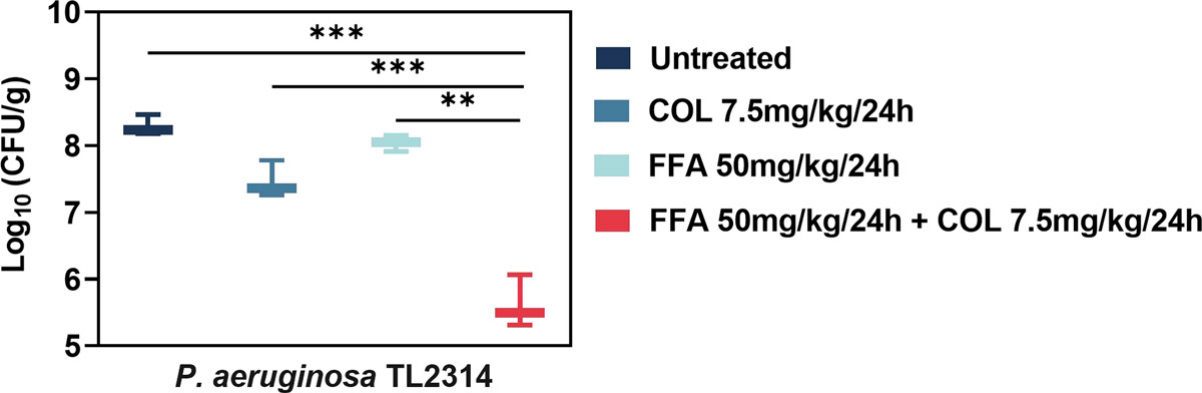
Quantification of bacterial loads 24 h following treatment with FFA and colistin alone or in combination in the thighs of mice infected with COL-R P. aeruginosa TL2314 (Δlog_10_ CFU/thigh/g). Data were analyzed by Student’s *t* test. **, *P* < 0.01; ***, *P* < 0.001.

A neutropenic mouse thigh infection model was also utilized to expand on these analyses. The administration of FFA (50 mg/kg of body weight) or colistin (7.5 mg/kg) alone inhibited P. aeruginosa TL2314 growth slightly 24 h after injection (Fig. 4). However, the combination of colistin and FFA (1.839-log_10_ CFU decrease compared with the colistin-alone treatment group) yielded superior efficacies compared with the single treatments (*P < *0.05). *In vivo* experiments showed that the combination of the two drugs had better antibacterial effects on COL-R P. aeruginosa TL2314 than single-agent treatments.

### Exploration of the mechanistic basis for the synergistic antimicrobial activity.

**FIG 5:**
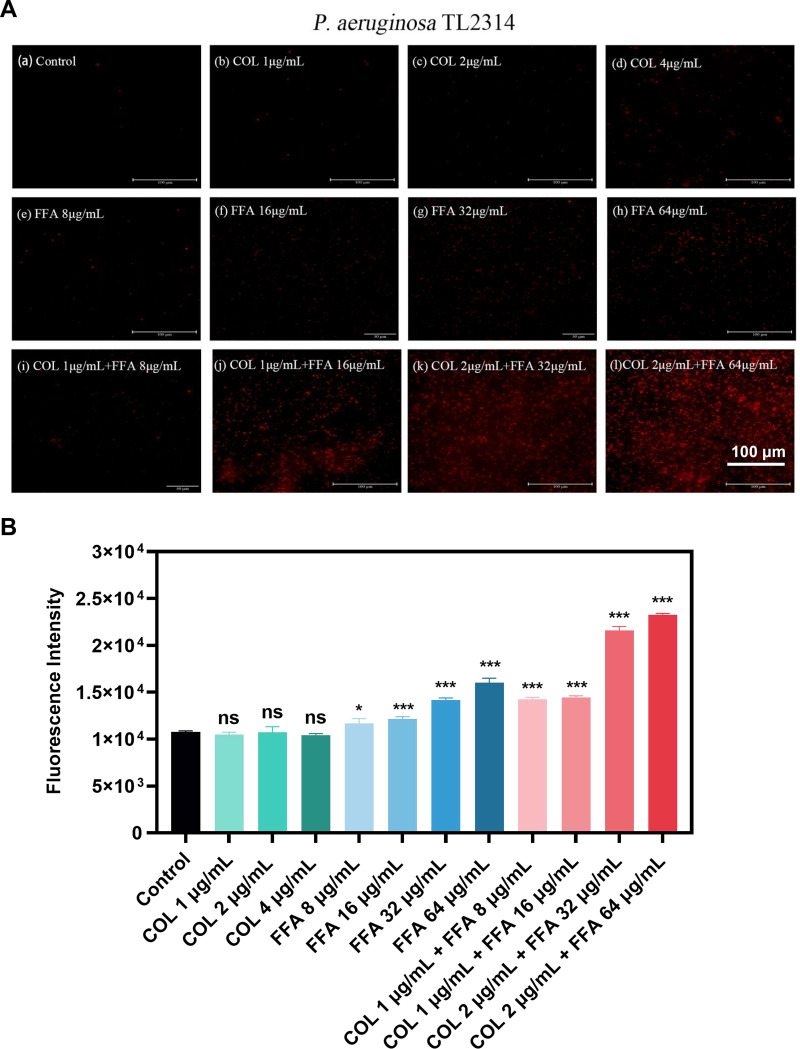
(A) Fluorescence images of P. aeruginosa TL2314 cells in the exponential phase of growth treated with colistin and/or FFA followed by incubation for 30 min with PI (50 μg/mL PI) before imaging. (B) Fluorescence intensity values for P. aeruginosa TL2314 cells in the exponential phase of growth treated with colistin and/or FFA followed by incubation for 30 min with PI (50 μg/mL PI). ns, not significant; *, *P* < 0.05; ***, *P* < 0.001 (by Student’s *t* tests). Data are means ± SD from triplicate analyses.

To explore the mechanisms governing the observed synergistic efficacy of combination FFA-colistin treatment, propidium iodide (PI), which binds to nucleic acids in bacteria with damaged membranes, was used to test the integrity of the membranes of TL2314 cells after treatment with these antimicrobial agents. Membrane permeability, as measured by the fluorescent PI signal, was not affected by colistin alone (1, 2, and 4 μg/mL) (Fig. 5A). However, preincubation with FFA led to a dose-dependent increase in the membrane permeability of these cells, consistent with the fluorescence microscopy images (Fig. 5B).

## DISCUSSION

Initially identified in 1947, colistin is a cationic cyclic peptide antibiotic that utilizes hydrophobic and polar interactions to disrupt the membrane permeability of Gram-negative bacteria, thereby killing these pathogens (16). Electrostatic interactions between positively charged colistin residues and negatively charged lipopolysaccharide moieties on the bacterial outer membrane are particularly important for such activity (17). The emergence of carbapenem-resistant P. aeruginosa, E. coli, K. pneumoniae, A. baumannii, and other bacterial strains has necessitated the use of colistin as a final bulwark against these MDR bacterial infections (18–21). However, the clinical value of colistin is limited by its potential to cause neurotoxicity and nephrotoxicity and by the emergence of colistin resistance, which is increasingly prevalent among Gram-negative clinical isolates (22). There is thus an urgent need for the design of reliable broad-spectrum antibacterial regimens that can lower the necessary colistin dosages and aid in the eradication of COL-R Gram-negative bacteria.

FFA is frequently used for the treatment of rheumatic disorders but can also suppress the growth of C. albicans and *S.* Typhimurium (12, 13). In recent reports, FFA has also been demonstrated to suppress the growth of MRSA and its ability to form biofilms (14). No previous reports have documented the antimicrobial or antibiofilm effects of FFA and colistin in combination when used to treat COL-R Gram-negative bacteria. Recent reports have documented a range of beneficial effects of FFA beyond its direct anti-inflammatory activity, including its ability to promote carbonyl reductase 1 (*CBR1*) upregulation and thereby protect against pulmonary damage induced by sepsis (23). It can also reportedly downregulate the expression of osteoclastogenesis-related genes while activating mitogen-activated protein kinase (MAPK) signaling and thereby preventing osteoporosis (24, 25), in addition to inducing the AMP-activated protein kinase (AMPK) activation and thereby enhancing angiogenic activity *in vitro* and *in vivo* (26). The pleiotropic properties of FFA may thus offer substantial benefits to treated patients.

Many recent reports have demonstrated that colistin can synergize with compounds other than antibiotics, providing an opportunity to better combat COL-R infections (27). The combination of nontraditional antibiotic compounds with antibiotics to form new treatment regimens has thus garnered considerable scientific attention. Accordingly, in this study, we documented synergistic antimicrobial efficacy when COL-R P. aeruginosa, E. coli, K. pneumoniae, and A. baumannii were treated with colistin and FFA. Notably, 90.3% (28/31) of the tested bacterial isolates exhibited MDR phenotypes in susceptibility assays, emphasizing the need to closely monitor the emergence of colistin resistance in an effort to prevent the widespread dissemination of these resistant strains. Using checkerboard and time-kill assays, combination FFA-colistin treatment was found to synergistically eradicate all tested bacterial isolates other than the TL1736, TL1744, and TL2967 strains, potentially owing to strain-specific differences (28).

In addition to preventing the establishment of new biofilms, combined FFA-colistin treatment was sufficient to eradicate mature biofilms *in vitro* in this study. SEM images revealed that relative to biofilms treated with FFA or colistin alone, the combination of these two drugs was sufficient to markedly disrupt biofilm structural characteristics relative to the intact structures observed for the control and single-agent treatment groups. Effectively treating infections caused by biofilms remains a pressing clinical challenge (29). These protective structures are composed of a variety of polysaccharides, lipids, nucleic acids, and proteins, and they can render these bacteria resistant to most antimicrobial treatment regimens (30). The ability of colistin and FFA to synergistically eradicate established biofilms highlights the promise of this antibacterial adjuvant regimen.

The *in vivo* efficacy of combination colistin-FFA treatment was tested with mouse and G. mellonella infection models. The combination of colistin and FFA significantly improved G. mellonella larval survival rates and reduced the bacterial burden detected in treated mice. Cytotoxicity analyses also confirmed the excellent biosafety profile of this combination treatment regimen. Analyses of the potential mechanism of action whereby FFA sensitizes cells to colistin were performed using membrane permeability assays with propidium iodide (PI), which showed that FFA may induce damage to the bacterial membrane, thereby enhancing the antibacterial efficacy of colistin (31–33). These findings demonstrated the potential of this combination for *in vivo* use in the future. Colistin can lead to both nephrotoxicity and neurotoxicity, and thus, its use has largely been discontinued. However, when combined with FFA, a markedly lower dose is required to achieve antibacterial efficacy, thereby minimizing the side effects associated with such treatment and underscoring the potential utility of this regimen *in vivo* in future studies and clinical trials.

Here, the combination of FFA and colistin was found to significantly enhance the sensitivity of COL-R Gram-negative bacteria to colistin, accompanied by the effective disruption of biofilms. However, further clinical trials will be necessary to validate the therapeutic value of this combined regimen. Even so, these findings emphasize the potential value of leveraging FFA as a means of restoring bacterial sensitivity to colistin, providing an opportunity to preserve the utility of colistin as an antibiotic of last resort for use when treating individuals suffering from infections caused by MDR Gram-negative bacteria. The major therapeutic implications of these findings warrant follow-up investigations to fully explore the potential clinical applications of FFA in combination with colistin or other antibiotics.

### Conclusion.

This study is, to our knowledge, the first to report the synergistic efficacy of combination FFA-colistin treatment as a means of eradicating COL-R Gram-negative bacteria and biofilms. These findings highlight the promise of this novel combined therapeutic regimen as an alternative strategy capable of addressing the growing clinical challenges posed by COL-R bacteria while also providing a foundation for future optimization-focused research efforts.

## MATERIALS AND METHODS

### Bacterial isolates.

A total of 31 nonduplicate COL-R Gram-negative bacterial isolates were selected randomly from the First Affiliated Hospital of Wenzhou Medical University, including COL-R P. aeruginosa (*n* = 8), E. coli (*n* = 8), K. pneumoniae (*n* = 8), and A. baumannii (*n* = 7) isolates. Bacterial identification was confirmed using a matrix-assisted laser desorption ionization–time of flight mass spectrometry (MALDI-TOF MS) (bioMérieux, Lyons, France) approach, and isolates were stored in Luria-Bertani (LB) broth supplemented with 30% glycerol at −80°C. E. coli ATCC 25922 and P. aeruginosa ATCC 27853 served as quality control isolates for this study.

### Antibiotic preparation.

FFA was obtained from Shanghai Yuanye Biotechnology Co., Ltd. (Shanghai, China), and stock solutions of 10 mg/mL were prepared by initially dissolving FFA in dimethyl sulfoxide (DMSO) and further diluting it with distilled deionized water to the concentration required for subsequent experiments (14). Colistin (COL), ciprofloxacin (CIP), aztreonam (ATM), cefepime (FEP), imipenem (IPM), gentamicin (GEN), levofloxacin (LVX), ceftazidime (CAZ), and tobramycin (TOB) were purchased from Wenzhou Kangtai Biotechnology Co., Ltd. Cation-adjusted Mueller-Hinton broth (CAMHB) was utilized for the antimicrobial test. All solvent selections and dilutions were performed according to the Clinical and Laboratory Standards Institute (CLSI) guidelines (34).

### Antimicrobial susceptibility testing.

A broth microdilution strategy was utilized to compute the MIC values of FFA and a range of common antibiotics when used to treat the 31 selected clinical COL-R bacterial isolates, as described in a previous report (35). Briefly, a 100-μL volume of the bacterial suspension was adjusted to a 0.5 McFarland standard, followed by transfer into the wells of 96-well plates containing 100-μL serial dilutions of FFA and the prepared antibiotics. Following a 16- to 18-h incubation at 37°C, the MIC values were determined by assessing the lowest antibiotic concentration sufficient to completely inhibit bacterial growth. Antimicrobial susceptibility testing results were interpreted based on the breakpoints in the CLSI 2021 guidelines. All analyses were repeated in triplicate.

### Checkerboard assays.

The *in vitro* synergistic efficacy of colistin and FFA was examined using a checkerboard assay approach using a modified version of a protocol employed in a previous report (36). Briefly, serial dilutions of FFA and colistin were mixed in 96-well plates to generate a range of dilution combinations. The *x* axis represents 2-fold serial dilutions of colistin (drug A) (from 128 μg/mL to 0.125 μg/mL), with the *y* axis representing 2-fold serial dilutions of FFA (drug B) (from 512 μg/mL to 8 μg/mL), to form a matrix. Individual bacterial suspensions were diluted with sterile saline to a 0.5 McFarland standard following culture overnight, and these suspensions were then diluted 1:100 using CAMHB. The resultant 100-μL bacterial suspension was added to the prepared 96-well plates containing 50 μL each of FFA and colistin to give a final bacterial concentration of 7.5 × 10^5^ CFU/mL. Results were examined after an incubation period of 16 to 20 h at 37°C.

Fractional inhibitory concentration (FIC) index values were calculated as follows: FIC(drug A) = (MIC of drug A in the combination)/(MIC of drug A alone), FIC(drug B) = (MIC of drug B in the combination)/(MIC of drug B alone), and FICI = FIC(drug A) + FIC(drug B). FICI values of ≤0.5, 0.5 to 4, and >4 indicate synergistic, indifferent, and antagonistic interactions, respectively (37). All analyses were repeated in triplicate.

### Time-kill assay.

A time-kill assay was carried out using COL-R P. aeruginosa, E. coli, K. pneumoniae, and A. baumannii strains (*n* = 2 each) selected at random to assess COL-R Gram-negative bacterial growth kinetics using a modified version of a previously reported protocol (38). Drug concentrations were selected from the above-described checkerboard analysis. Treatment groups included control (untreated), FFA (8, 32, and 64 μg/mL), colistin (0.125, 0.5, 1, and 2 μg/mL), and combination (colistin plus FFA) groups. Briefly, 200 μL of prepared bacterial suspensions at a 0.5 McFarland standard were added to 20 mL of LB broth containing the indicated treatments in a 50-mL centrifuge tube. After 2, 4, 6, 12, and 24 h, bacterial suspensions were collected from these tubes and subjected to serial 10-fold dilution, with 100 μL of these dilutions being spread onto LB agar plates, which were incubated at 37°C overnight before colonies were counted. Bactericidal activity was defined as a ≥3-log_10_ decline in the bacterial CFU per milliliter over this 24-h incubation period, with synergistic bactericidal activity being defined as a ≥2-log_10_ decline in the bacterial CFU per milliliter relative to the most active single-agent treatment dose (39). All analyses were repeated in triplicate.

### Analysis of the inhibition of biofilm formation.

Crystal violet staining was used to assess the inhibition of biofilm formation based on a modified version of a previously reported protocol (40). The bacteria were inoculated onto blood agar plates and incubated overnight, after which individual colony isolates on the plates were adjusted to a 0.5 McFarland standard, diluted 1:100 with fresh LB broth, and added to 96-well plates (100 μL/well). FFA and colistin were diluted to the appropriate concentrations selected from the above-described checkerboard analysis in LB broth and added to each well alone or in combination as appropriate in a final volume of 100 μL. After a 24-h incubation at 37°C, the medium was poured out of these wells, which were rinsed two times using 200 μL of 1× phosphate-buffered saline (PBS) to remove any planktonic bacteria. The plates were allowed to air dry at room temperature, after which they were stained for 15 min at 37°C with 250 μL of 1% crystal violet. After staining, the 96-well plates were washed three times with 200 μL of 1× PBS. Next, the plates were naturally dried, and 200 μL of 95% ethanol–5% acetic acid was added to dissolve the crystal violet. The optical density at 595 nm (OD_595_) was measured on a microplate reader (Multiskan FC). The experiment was repeated three times.

### Mature biofilm eradication assay.

A slightly modified version of a previously reported protocol was used to test the ability of colistin and/or FFA to eradicate established bacterial biofilms (41). Briefly, bacterial suspensions were prepared as described above, and 200 μL was added to each well of a 96-well plate. Following 24 h of static incubation at 37°C to allow the formation of mature biofilms, the supernatants were discarded, and the plates were washed three times with 0.9% saline to remove the unattached cells. Next, FFA and colistin were added to the 96-well plates at final concentrations of 128 μg/mL and 8 μg/mL, respectively, either alone or in combination, and incubated at 37°C for 24 h. The dyeing procedure is the same as the one described above. All analyses were repeated in triplicate.

### Scanning electron microscopy.

Biofilms of P. aeruginosa TL2314 were observed by scanning electron microscopy to facilitate in-depth analyses of the effects of colistin and/or FFA, using a slightly modified version of a previously reported protocol (42). These bacteria were cultured overnight and diluted to a 0.5 McFarland standard, and sterile coverslips were added to individual wells of 6-well plates. For each of the four tested conditions (untreated and colistin, FFA, and FFA-colistin treated), 100 μL of the prepared bacteria was added to each well along with 1,900 μL of LB broth containing FFA (32 μg/mL), colistin (0.5 μg/mL), or both. The plates were incubated for 18 to 24 h at 37°C, after which the coverslips were rinsed using PBS. Biofilms were then fixed with 2.5% glutaraldehyde (4 h at 4°C) and dehydrated with a serial ethanol dilution series (15 min each of 20%, 40%, 70%, 90%, 95%, and 100% [vol/vol]). SEM analyses were performed using an S3000N instrument (Hitachi, Tokyo, Japan) based on the directions provided by the manufacturer.

### *In vitro* analyses of cytotoxicity.

RAW264.7 cells (ATCC, Manassas, VA, USA) were cultured to confluence in Dulbecco’s modified Eagle’s medium (DMEM) supplemented with 10% heat-inactivated fetal bovine serum (FBS) in a CO_2_ incubator at 37°C. Cells were harvested with trypsin and transferred into 96-well plates (1 × 10^5^ cells/well in 100 μL). After a 24-h incubation, 10-μL serial dilutions of FFA (4, 8, 16, 32, 64, and 128 μg/mL) or combinations of FFA and colistin were added to individual wells. After an additional 24-h incubation, 10 μL of cell counting kit 8 (CCK-8) reagent (Solarbio, Beijing, China) was added to each well. Following a 1-h incubation at 37°C in the dark, the OD_450_ was measured on a microplate reader (Multiskan FC). FBS containing DMSO (the solvent used for FFA) as a vehicle control was used as a negative control (43). Three duplicates of every analysis were performed.

### Galleria mellonella infection model.

A modified version of a previously reported G. mellonella infection model system was used to assess the *in vivo* synergistic efficacy of FFA and colistin (44). Briefly, TL2314, DC7333, FK6556, and BM2431 were cultured overnight and then diluted to 1.5 × 10^5^ CFU/mL. Treatment groups in this assay included control (untreated), colistin (0.125, 0.5, 1, and 2 μg/mL), FFA (8, 32, and 64 μg/mL), and combination colistin-FFA groups (*n* = 10/group). A sterile saline solution was used to treat control larvae. Larvae (250 to 350 mg) were randomly assigned to individual groups, and a 10-μL volume of the prepared bacterial solution was injected with a microinjector into the rear left proleg of each G. mellonella specimen, with 10 μL of appropriate antibiotic treatments being injected 2 h after bacterial inoculation. G. mellonella survival rates were recorded after 24, 48, 72, 96, 120, 144, and 168 h. When larvae persistently failed to react to physical stimulation, they were regarded as dead. Survival rates were assessed with Kaplan-Meier curves and log rank tests. All analyses were repeated in triplicate.

### *In vivo* analyses of synergistic antimicrobial efficacy in mice.

A neutropenic mouse thigh infection model was established to facilitate further analyses of the *in vivo* efficacy of colistin and FFA. Female BALB/c mice (5 to 6 weeks old; Charles River, Hangzhou, China) were utilized in a manner consistent with Laboratory animal - Requirements of environment and housing facilities (GB 14925-2010) (45). The Zhejiang Association for Science and Technology SYXK approved these analyses (identifier SYXK [Zhejiang] 2018-0017), and these analyses were performed according to Laboratory animal—Guideline for ethical review of animal welfare (GB/T 35892-2018) (46).

To induce neutropenia (≤100 neutrophils per mm^3^), mice were intraperitoneally injected with cyclophosphamide (Yuanye Biotechnology Co., Ltd., Shanghai, China) 4 days (150 mg/kg) and 1 day (100 mg/kg) prior to the establishment of a thigh infection model. P. aeruginosa TL2314 was selected at random as a model COL-R strain, and a 100-μL suspension of these bacteria in the exponential phase of growth (1.5 × 10^7^ CFU/mL) was injected into the posterior thigh muscle of each mouse. Two hours following bacterial injection, mice were intraperitoneally administered colistin (7.5 mg/kg) alone or together with FFA (50 mg/kg) (47). After 24 h, the thigh muscles of the mice were removed under sterile conditions, deboned, and weighed. The tissue was then cut into small pieces and placed in a tissue-grinding tube, after which 1 mL of normal saline and two grinding beads were added, and the contents were ground in a tissue grinder (KZ-III-F; Servicebio, Hubei, China) for an appropriate time and frequency until the tissue was homogenized. The thigh muscle homogenate was serially diluted 10 times, and 100-μL aliquots of the dilutions were spread onto Trypticase soy agar plates to quantify the bacterial titers in the different drug treatment groups (untreated, colistin alone, FFA alone, and the combination). The bacterial burden was quantified by counting the log_10_ CFU per gram from the thigh muscle homogenates. Groups of 3 mice (6 thigh infections) were included for each dosing regimen.

### Membrane permeability assay.

Membrane permeability was assessed using a modified version of a previously reported protocol (48). Briefly, TL2314 cells in the exponential phase of growth were treated with either FFA (8, 16, 32, and 64 μg/mL), colistin (1, 2, and 4 μg/mL), or a combination of the two for 2 h. Cells were then stained for 30 min at 37°C with propidium iodide (PI) (50 μg/mL). The fluorescence intensity was then assessed with a microplate reader (BioTek) (excitation at 535 nm and emission at 615 nm) or a fluorescence microscope (Nikon, Japan). All analyses were repeated in triplicate.

### Statistical analysis.

Data are means ± standard deviations (SD) from three or more replicate trials. Data were compared with two-sample *t* tests using GraphPad Prism 8.0. Statistical significance is indicated by asterisks in the figures (*, *P* < 0.05; **, *P* < 0.01; ***, *P* < 0.001).
