# Supplementary material for: Flufenamic Acid, a Promising Agent for the Sensitization of Colistin-Resistant Gram-Negative Bacteria to Colistin
Source: Microbiol Spectr. 2023 Mar 27;11(2):e04052-22. doi: 10.1128/spectrum.04052-22 (PMC10100705; doi:10.1128/spectrum.04052-22)
Supplement: Supplemental file 1 — Supplemental material. Download spectrum.04052-22-s0001.pdf, PDF file, 0.3 MB [file spectrum.04052-22-s0001.pdf]

Table S1 Patient's clinical data and characteristics of analyzed strains.

Species	Strains	Isolation Date	Age	Gender	Sample	Ward
P. aeruginosa	TL1671	11/04/2015	74	M	Wound	Endocrinology
	TL1736	20/05/2015	44	M	Sputum	Neurosurgery
	TL1744	23/05/2015	63	M	Sputum	ICU
	TL2314	08/03/2016	66	M	Sputum	ICU
	TL2917	09/02/2017	58	M	Sputum	ICU
	TL2967	13/03/2017	26	M	Sputum	Emergency
	TL3008	05/04/2017	67	M	Sputum	Neurosurgery
	TL3086	24/05/2017	65	M	Sputum	Neurosurgery
E. coli	DC90	27/03/2012	76	M	Wound	Gastrointestinal Surgery
	DC3539	19/1/2015	74	M	Sputum	Neurosurgery
	DC3599	13/04/2015	72	M	Sputum	Respiratory
	DC3806	19/05/2015	17	F	Sputum	Hematology
	DC3846	28/05/2015	77	F	Urine	Urology
	DC4887	23/02/2016	63	M	Urine	Urology
	DC5286	23/05/2016	82	F	Urine	Endocrinology
	DC7333	18/05/2016	32	M	Drainage fluid	ICU
K. pneumoniae	FK169	02/07/2012	79	F	Sputum	General
	FK1342	06/07/2014	21	F	Fester	Gastrointestinal Surgery
	FK1913	19/01/2015	74	M	Sputum	Neurosurgery

	FK1986	03/03/2015	44	M	Blood	Endocrinology
	FK3810	17/03/2017	21	F	Stool	ICU
	FK3994	19/05/2017	69	M	Sputum	Nephrology
	FK6556	02/11/2015	68	M	Sputum	CCU
	FK6663	04/05/2019	73	M	Stool	ICU
	BM1539	13/02/2014	48	M	Sputum	ICU
	BM1595	03/04/2014	57	F	Sputum	ICU
	BM2349	17/02/2015	68	F	Sputum	ICU
A. baumannii	BM2370	28/02/2015	59	F	Sputum	ICU
	BM2412	17/03/2015	63	F	Sputum	ICU
	BM2431	24/03/2015	89	M	Sputum	ICU
	BM2622	02/06/2015	59	M	Sputum	RICU

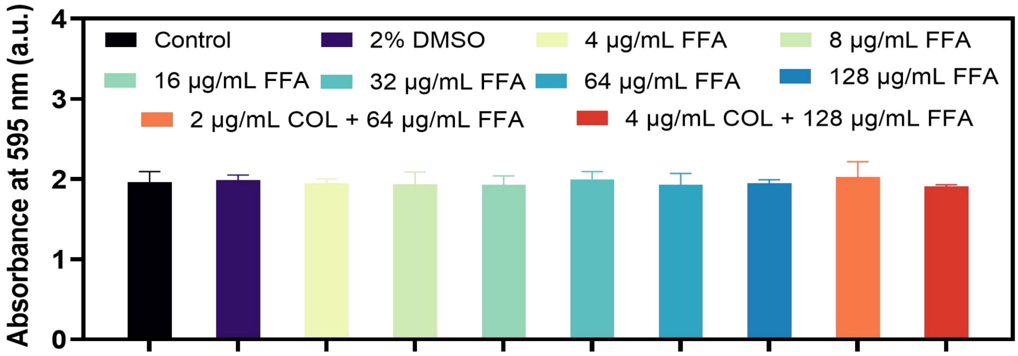

Figure S1 Cytotoxicity of different FFA concentrations and DMSO (2%) measured in RAW264.7 murine macrophages. Data were analyzed using Student's t-test and represent means ± SD from triplicate analyses. DMSO: dimethyl sulfoxide.
